# Draft Genome Sequences of Six Yersinia pestis Strains Isolated from a Natural Plague Focus in Mongolia

**DOI:** 10.1128/MRA.00831-20

**Published:** 2020-10-22

**Authors:** Jing Wang, N. Tsogbadrakh, Jingliang Qin, Hua Yun, D. Ganbold, Guangyu Zhao, Yujun Cui, Sheng Zhang

**Affiliations:** a Chinese Academy of Inspection and Quarantine, Beijing, China; b National Center for Zoonotic Diseases, Ulaanbaatar, Mongolia; c State Key Laboratory of Pathogen and Biosecurity, Beijing Institute of Microbiology and Epidemiology, Beijing, China; d Huhhot Customs District, Huhhot, China; Broad Institute

## Abstract

In this announcement, we report the draft genome sequences of six Yersinia pestis strains (biovar Medievalis) that were isolated from the Zamyn-Ude region in Mongolia. These genomes reveal the genetic characteristics of the Y. pestis population circulating in a local plague focus.

## ANNOUNCEMENT

Yersinia pestis, a Gram-negative bacillus, is established in hundreds of natural plague foci around the world, where it causes sporadic epizootics, primarily in rodents (1), and has caused three major pandemics in history (2). Humans are at the greatest risk of exposure to Y. pestis during plague epizootics, because mass rodent die-offs increase the potential for human exposure to sick or dead animals and infected fleas (3). In recent years, plague has frequently occurred in many provinces and counties in Mongolia (4).

The Zamyn-Ude natural plague focus is located in Dornogovi Province, at the border between Mongolia and China. The six Y. pestis strains were isolated from the Zamyn-Ude region during the 2018 coinvestigation of the vectors, reservoirs, and pathogens in the natural foci of zoonotic infectious diseases in the selected border areas of Mongolia and China. All strains belong to the biovar Medievalis (5). Y. pestis strains were isolated from dead rats in the Zamyn-Ude natural plague focus (43.73N, 111.89E) in 2018, cultured on Hotinger's agar (pH 6.9 to 7.1; prepared in the National Center for Zoonotic Diseases, Mongolia) at 28°C for 2 days by bacteriological methods, and identified with microscopic examinations (6), PCR methods, and a biochemical identification test (7). The Qiagen DNeasy blood and tissue kit was used to extract DNA samples.

The sequencing libraries were prepared using the MGIEasy universal DNA library preparation set (BGI, Shenzhen, China), and sequencing was performed on the Illumina NovaSeq 6000 sequencing platform, with a 150-bp paired-end library constructed according to the manufacturer’s instructions. We obtained 27,740,766 to 44,115,814 reads for each genome. The library insert size was 350 bp, and more than 0.9 Gb of clean data were generated for each strain. Adapter sequences and low-quality sequencing reads (Q, <20) were trimmed with Trimmomatic v0.38 (8) using the following options: ILLUMINACLIP:TruSeq3 PE 2.fa:2:30:10; LEADING:5; TRAILING:5; SLIDINGWINDOW:5:20; and MINLEN:50. After the raw data were filtered, we obtained more than 178 Mb of clean reads for each strain, and the average sequencing depth was 86×. SPAdes v3.12.0 (http://cab.spbu.ru/software/spades) (9) was used to perform the assembly with default parameters. Finally, we obtained assembled genomes with an average genome size of 4.6 Mb. The GC content of the genomes is 47.55%. Each genome contains 202 to 224 contigs (Table 1). The coding sequences of the six isolates were annotated using the NCBI Prokaryotic Genome Annotation Pipeline (PGAP) (10).

**TABLE 1:** Information on the Y. pestis strains

Strain name	SRA accession no. (raw data)	GenBank accession no.	No. of contigs	Avg sequencing depth (×)	Size (bp)	Avg length (bp)	*N*_50_ (bp)	*N*_90_ (bp)	Total no. of predicted genes	GC content (%)
NCZD3304	SRR10961165	JAAEFC000000000	219	86	4,635,546	18,542.18	47,081	13,226	4,130	47.55
NCZD3305	SRR10961164	JAAEFB000000000	213	86	4,631,355	19,624.39	45,252	13,570	4,130	47.55
NCZD3306	SRR10961163	JAAEFA000000000	224	86	4,637,696	18,258.65	46,702	13,226	4,133	47.55
NCZD3307	SRR10961162	JAAEEZ000000000	202	86	4,626,934	21,127.55	46,702	13,669	4,122	47.55
NCZD3309	SRR10961161	JAAEEY000000000	223	86	4,634,050	19,635.81	47,081	13,568	4,137	47.55
NCZD3310	SRR10961160	JAAEEX000000000	200	86	4,627,744	21,726.5	47,079	14,238	4,120	47.55

Ethical approval was not required because these strains were isolated from dead rats.

### Data availability.

The draft genome assemblies reported here are available in GenBank under the accession numbers JAAEFC000000000, JAAEFB000000000, JAAEFA000000000, JAAEEZ000000000, JAAEEY000000000, and JAAEEX000000000. All raw sequencing reads have been deposited in the NCBI under the BioProject number PRJNA602786 (Table 1) and are available in the Sequence Read Archive (SRA).
